# Is MRI-Linac helpful in SABR treatments for liver cancer?

**DOI:** 10.3389/fonc.2023.1130490

**Published:** 2023-03-16

**Authors:** Agnès Tallet, Jean-Marie Boher, Marguerite Tyran, Hugues Mailleux, Gilles Piana, Mohamed Benkreira, Pierre Fau, Naji Salem, Laurence Gonzague, Claire Petit, Julien Darréon

**Affiliations:** ^1^ Department of Oncology Radiation Therapy, Institut Paoli-Calmettes, Marseille, France; ^2^ Centre de Recherche en Cancérologie de Marseille, Unité Mixte de Recherche (UMR1068), Marseille, France; ^3^ Department of Biostatistics, Institut Paoli-Calmettes, Marseille, France; ^4^ Department of Medical Physics, Institut Paoli-Calmettes, Marseille, France; ^5^ Department of Radiology, Institut Paoli-Calmettes, Marseille, France

**Keywords:** MRI-Linac, liver, SAbR, CBCT, conventional Linac

## Abstract

**Objective:**

To determine the MRI-Linac added value over conventional image-guided radiation therapy (IGRT) in liver tumors Stereotactic ablative radiation therapy (SABR).

**Materials and methods:**

We retrospectively compared the Planning Target Volumes (PTVs), the spared healthy liver parenchyma volumes, the Treatment Planning System (TPS) and machine performances, and the patients’ outcomes when using either a conventional accelerator (Versa HD®, Elekta, Utrecht, NL) with Cone Beam CT as the IGRT tool or an MR-Linac system (MRIdian®, ViewRay, CA).

**Results:**

From November 2014 to February 2020, 59 patients received a SABR treatment (45 and 19 patients in the Linac and MR-Linac group, respectively) for 64 primary or secondary liver tumors. The mean tumor size was superior in the MR-Linac group (37,91cc vs. 20.86cc). PTV margins led to a median 74%- and 60% increase in target volume in Linac-based and MRI-Linac-based treatments, respectively. Liver tumor boundaries were visible in 0% and 72% of the cases when using CBCT and MRI as IGRT tools, respectively. The mean prescribed dose was similar in the two patient groups. Local tumor control was 76.6%, whereas 23.4% of patients experienced local progression (24.4% and 21.1% of patients treated on the conventional Linac and the MRIdian system, respectively). SABR was well tolerated in both groups, and margins reduction and the use of gating prevented ulcerous disease occurrence.

**Conclusion:**

The use of MRI as IGRT allows for the reduction of the amount of healthy liver parenchyma irradiated without any decrease of the tumor control rate, which would be helpful for dose escalation or subsequent liver tumor irradiation if needed.

## Introduction

Liver tumor Stereotactic ablative radiation therapy (SABR) is particularly challenging because these tumors cannot be individualized from the healthy liver parenchyma with the use of conventional image-guided radiation therapy (IGRT) tools (Cone Beam Computed Tomography, CBCT), requiring fiducial markers implantation, and are breath motion-dependent. The implantation of fiducial markers, which is an invasive step, helps in tumor localization but fails to inform about tumor boundaries, and a geographic missing is always of concern. The issue of tumor motion during the radiation delivery is managed by using compression devices and also simulation techniques collecting the various tumor positions all along a breathing cycle (1). Nonetheless, this approach suggests to extent tumor margins, and therefore, to irradiate a larger width of healthy liver tissue. Improving IGRT techniques is a way to better shrinking the PTV around the tumor and may involve gating techniques, magnetic resonance imaging (MRI) use, or both. MRI allows the visualization of the exact tumor, with the advantage of avoiding the fiducial markers invasive step, with its rare but potential side-effects, as well as its possible inaccuracy under some circumstances (2). Moreover, it might lead to better local control rates by overcoming the geographic-missing issue. MRI is well-known for its high resolution in soft tissue. Therefore, MR-guided-radiation therapy (MRgRT) is assumed to improve tumor localization while directly tracking the tumor throughout the treatment session, allowing the safe reduction of tumor margin size. Moreover, the MR-Linac includes a comprehensive process of online adaptive radiation therapy with daily-optimized dosimetry, making it possible to safely treat lesions close to radiosensitive organs (such as the stomach or the duodenum).

We implanted MRgRT in our radiation therapy department in early 2019 (MRIdian®, ViewRay, CA). Before this date, liver SABR treatments were performed on a conventional linear accelerator (Linac) using a 6 MV Flattening Filter Free beam with CBCT as IGRT (Versa HD®, Elekta, Utrecht, NL).

To assess the added value of MRgRT in liver SABR, we herein compare the tumor visibility, the planning target volume (PTV) sizes, the treatment planning system (TPS) and machine performances, as well as tumor control and treatment toxicity, in patients treated on a conventional Linac or the MRIdian system.

## Materials and methods

We retrospectively gathered and reviewed the charts of patients with liver tumors treated by SABR from November 2014 to February 2020. From 2014 to 2018, patients were treated on the VERSA Linac with conventional IGRT (group CBCT), while from 2019 and onward, they were treated on the MRIdian system (group MRI). All SABR treatments were decided in a multidisciplinary discussion. Eligible patients were those with a Karnofsky Performance Status of more than 70%, no more than three lesions, with a maximum cumulative size of less than 60 mm, a Child-score A or B, and suitable biological liver-function, defined as hepatic-enzymes count inferior to three times the usual-values and a prothrombin time >70. Liver tumors were histologically proven either by previous surgery or by biopsy.

MRgRT advantages are theoretically multiple, assumed to contribute to both better local tumor control and lesser adverse events. First, target delineation is directly performed on the MRI-simulation scan, avoiding any image registration potential inaccuracy and subsequent geographic missing; second, the MRIdian system allows for target visualization and gating during the fraction, assuring the dose delivered strictly to the target for the whole fraction. These two perks are supposed to improve local control. Third, the MRIdian system has a unique double-stacked collimator allowing for a sharp dose fall-off, reducing the amount of healthy tissue irradiated; fourth, online adaptive radiation therapy allows for daily dosimetric optimization, better sparing Organs-at-risk from the radiation therapy dose; both are being supposed to reduce radio-induced side effects.

### Simulation

For Linac-based treatments, patients were first referred to the interventional radiology department for three fiducial markers implantation surrounding the tumor, at least one week before the simulation-CT. Immobilization devices consisted of a dedicated vacuum ball mattress and an abdominal compression system. For images acquisition, patients were lying in the supine position, arms above the head, immobilized in the Elekta BodyFix^®^ system with abdominal compression, before a first CT-scan (GE Optima^®^ CT 580 RT scanner), generating 2.5 mm axial images from two centimeters above diaphragmatic domes up to L4-L5, was acquired in free-breathing. When contrast enhancement was required, we performed two CT-scan (venous-time and arterial-time) for cholangiocarcinoma or hepatocellular carcinoma and one CT-scan (venous time) for liver metastases. Without 4DCT, rapid and repeated axial CT acquisitions were performed to provide a spatial sampling of target position and fiducial markers over multiple respiratory cycles. A 3mm-slice thickness MIP (Maximum Intensity Projection) reconstruction (1), every 3mm is performed with the axial acquisitions. Then motion margins were obtained after image registration between CT-scan and MIP by analyzing the fiducial marker deformation. Briefly, MIP reconstruction gives information on the maximum gray value in a CT reconstruction slice and the deformation of the fiducial marker, which allows us to deduce the information of motions margins to add.

For MRgRT-based treatments, we avoided the fiducial markers implantation step. The immobilization device only consisted of a simple MR-compatible all-fits-one contention device. The unique MRI sequence used was a fast imaging sequence, true fast-imaging with steady-state free precession (TRU-FISP), allowing the reduction of motion artifacts. The simulation was based-on a free-breathing CT-scan (for electron density calculation purpose, same as those performed for Linac-based treatments) and both an early-inhale breath-hold and a free-breathing MRI-scan in the supine position, arms above the head, acquired in 17 seconds. The MRI scan could be repeated to obtain the best image definition, the less blurring and with little motion artifacts, to be used for the treatment planning.

### Target volumes definition

For Linac-based treatments, tumor delineation was performed either directly on the simulation CT-scan, or more often, by using simulation-CT scan registration with the diagnosis-MRI or the (18)Fluorodeoxyglucose PET (FDG-PET). The gross tumor volume (GTV) was expanded according to the MIP to generate an internal target volume (ITV). The planning target volume (PTV) was obtained by adding to the ITV 5mm laterally and A/P and 7mm up and down.

For MRgRT-based treatments, the GTV was directly delineated on the MRI-simulation scan, using diagnosis-MRI as control. When tumor visualization was weak, the SABR treatment was maintained on the MRIdian system only if a close and highly identifiable liver structure indisputably enabled its localization. PTV was obtained by isotropically expanding the GTV by 5 mm or 3 mm for tumors poorly or accurately distinguishable from the liver parenchyma, respectively.

### Dosimetry and Dose prescription

#### Planning on the VERSA-HD

Treatment plans were generated on CT-scan images using the Collapsed Cone dose engine of the RayStation^®^ 8A (RaySearch^®^) Treatment Planning System (TPS). An inverse planning volumetric-modulated arc therapy (VMAT) technique was used, with two full-coplanar arcs of 6 MV photons FFF delivered with an Elekta Versa HD^®^ linear accelerator equipped with an Agility^®^ multi-leaf collimator (MLC) of 160 leaves of 5 mm width at a source to axis distance (SAD) of 100 cm. During the optimization process, the leaf speed was limited to 0.05 cm/degree to reduce the interplay effect, as shown by Court et al. (3). The median dose prescribed to the PTV was 40Gy [35–50] in 5 fractions [3–10].

#### Planning on the MRIdian system

The treatment plans used MR images, mostly with voluntary moderate inspiration breath-hold. CT images of the patient in the same position were co-registered to get electron density values for each voxel of the calculation grid. Dose calculation used the Monte-Carlo dose engine of the MRIdian^®^ (ViewRay^®^) TPS, taking into account the presence of the 0.35T magnetic field. We used an inverse-planning intensity-modulated radiation therapy (IMRT) step-and-shoot technique, with around 70 segments and 20 beams of 6MV photons FFF, delivered with the MRIdian^®^ system, equipped with a 138-leaf double-focused double-stacked MLC. The double-stacked MLC uses an offset between the top and bottom stack to achieve an effective leaf-width of 4.15 mm at 90 cm SAD. The dose rate was 650 MU/min. The treatment was delivered either in free-breathing or in early-inhale breath-hold using the respiratory gating functionality of the MRIdian, with the percentage of PTV allowed outside the 5mm-gating boundaries set at 10%.

For both groups, we used a calculation grid of 2 mm, and the dose was prescribed on the near-minimum-dose (98%) of the PTV.

Organs at risk dose constraints fulfilled Timmerman’s recommendations (4).

### Tumor visibility

**Figure 1:**
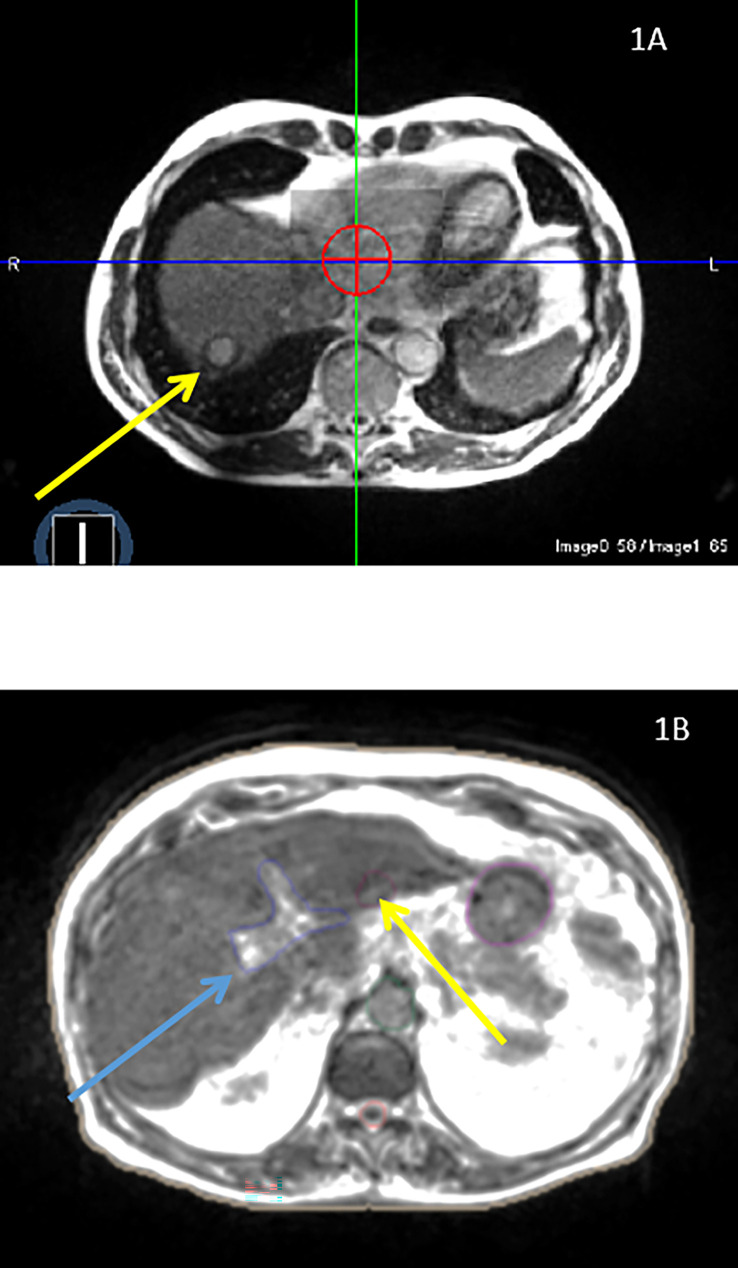
**(A)** A 69-years old patient presenting with single liver metastasis from pancreas located in the liver segment V that is easily visible. **(B)** A 78-years old patient presenting with a recurrent hepatocellular carcinoma. The primary liver tumor, located in the liver segment III (yellow arrow), is only visible on the diagnosis MRI after contrast injection and, therefore, requires the use of an adjacent structure (left portal vein, blue arrow) for accurate patient positioning.

A crucial advantage of the MRgRT, as compared to CBCT, is to allow direct tumor visualization on positioning images. Although tumor visualization is a highly subjective characteristic, its assessment had an informative purpose. The same radiation oncologist retrospectively retrieved this information for all cases, defining it either as accurately or poorly visible (**Figures 1A, B**, respectively). Poorly-visible tumors were those requiring the use of other hallmark structures in the close vicinity.

### Statistics

This retrospective study was approved by the institutional review board committee.

#### Volumes comparison

Tumor volumes were delineated on the simulation CT-scan or MRI-scan, with or without the help of co-registered diagnosis images, by the same radiation oncologist.

We compared the PTV size generated by each planning technique, as well as the volume expansion from the GTV. Dosimetry metrics, including PTV and radiation-spared healthy liver (liver – PTV) minimum, mean, and maximum doses, V40Gy, V30Gy, V20Gy, V15Gy, V5Gy-isodoses, and the conformation number (CN) (5), were calculated and compared between both techniques by the Log Rank test. Local tumor control rates at twelve months were assessed with 95% confidence intervals using the Kaplan-Meier method.

As TPS and treatment delivery modes are different in the two studied techniques, we assessed the treatment delivery systems’ performance by comparing in each patient treated on the MRIDian system the dosimetry results with those fictitiously generated for the Versa-HD machine. Using the CT-scan performed during MRgRT-based treatments simulation, VMAT treatments have been planned for each patient from the MRI group, using the same PTV as the one used in MRgRT treatment. Comparisons among groups were performed using Wilcoxon signed rank test for paired data.

#### Patient outcomes

Patients were followed by liver MRI and CT-scan every three months. As stated by the RECIST criteria, a complete response was defined by the total disappearance of the irradiated lesion; partial response corresponded to a ≥30% tumor regression; stable disease was declared by a ≤30% or ≤20% tumor regression or progression, respectively, whereas tumor progression was declared when tumor exceeded 20% of the initial volume (**Table 1**). Toxicity was prospectively scored using the Cancer Institute Common Terminology Criteria for Adverse Events Version 4. Common liver SABR toxicity includes nausea or vomiting, abdominal pain, duodenal or gastric ulcer or perforation, and radio-induced liver disease, all of which were carefully investigated during patient follow-up.

**Table 1:** Liver SABR toxicity in the CBCT and the MRI groups.

Toxicity type	CBCT group (45 tumors)	MRI group (19 tumors)
Nausea (n, %)		
Grade 1	7 (16%)	2 (11%)
Grade ≥2	0	0
Abdominal pain (n, %)		
Grade 1	3 (7%)	1 (5%)
Grade 2	0	1 (5%)
Grade ≥3	0	0
Gastritis (n, %)		
Grade 1	1 (2%)	0
Grade 2	1 (2%)	0
Grade ≥3	0	0
Gastric hemorrhage		
Grade 1	0	0
Grade 2	0	0
Grade 3	1(2%)	0
Grade ≥4	0	0
Duodenal Ulcer (n, %)		
Grade 1	0	0
Grade 2	1 (2%)	0
Grade ≥3	0	0
Radio-induced liver disease (n, %)		
Grade ≤4	0	0
Grade 5	1 (2%)	0
Portal hypertension		
Grade 3	0	1 (5%)
Hepatobiliary disease (n, %)	0	0

## Results

From November 2014 to February 2020, 59 patients were treated by SABR for 64 primary (9 patients) or secondary (n= 55) liver tumors; 41 and 18 patients were treated on the VERSA-HD FFF Linac (CBCT group) and the MRIdian system (MRI group), respectively. Patient characteristics are detailed in **Table 2** and were quite similar between the two groups.

**Table 2:** Patient characteristics.

	VERSA-HD	MRIdian	p-value*
n	45	19	
Age, median (range)	69 (30-83)	70 (38-89)	*0.974*
Tumor size, mean (SD)	20.86cc(22.94)	37.91cc (50.40)	*0.467*
Liver primary, n (%)	5 (11%)	4 (21%)	* *
Liver metastases, n (%)			
Colorectal	17 (38%)	8 (42%)	0.5357
Breast	8 (18%)	4 (21%)	
other	15 (33%)	3 (16%)	
Nb of metastases, n (%)			1.000
1	38 (90%)	17 (94%)	
2	4 (10%)	1 (6%)	
Mean dose			0.7573
Mean (SD) Median	42.21 (4.23) 40	41.79 (4.12) 40	
Nb of fractions	5.96 (2.06) 5.0	6.32 (2.26) 5.0	0.6061
Primary controlled			*0.3136*
Yes	38 (84%)	14 (74%)	
no	7 (16%)	5 (26%)	
Active metastatic disease			*0.7458*
Yes	17 (38%)	8 (42%)	
no	28 (62%)	11 (58%)	
Previous liver tumor-directed therapy			*0.9448*
No	19 (42%)	8 (42%)	
One previous treatment	11 (25%)	4 (21%)	
Several previous treatments	15 (33%)	7 (37%)	
First metastasis (or liver tumor)			*0.6555*
Yes	14 (31%)	7 (37%)	
no	31 (69%)	12 (63%)	

### Tumor visibility on IGRT

**Figure 2:**
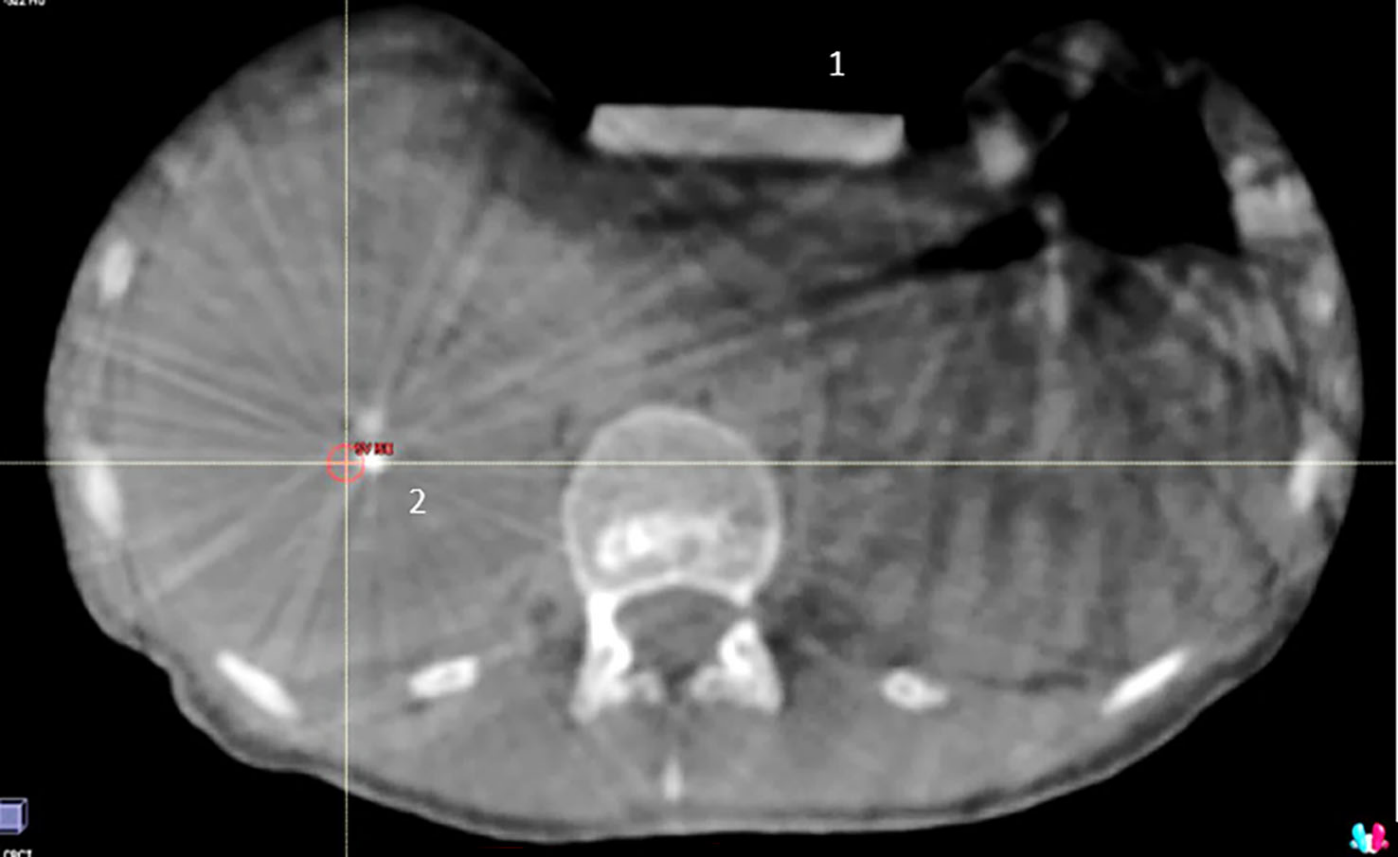
1: compression system; 2: fiducials markers generating artifacts; tumor is not visible due to both poor image resolution and fiducial markers artifacts.

As expected, CBCTs never allowed tumor boundaries visualization and only prior fiducial markers implantation made patient positioning feasible (**Figure 2**). Conversely, liver tumor borders were accurately and poorly identified on the TRU-FISP MRI sequence in 72% and 28% of cases, respectively.

### PTV size

**Figure 3:**
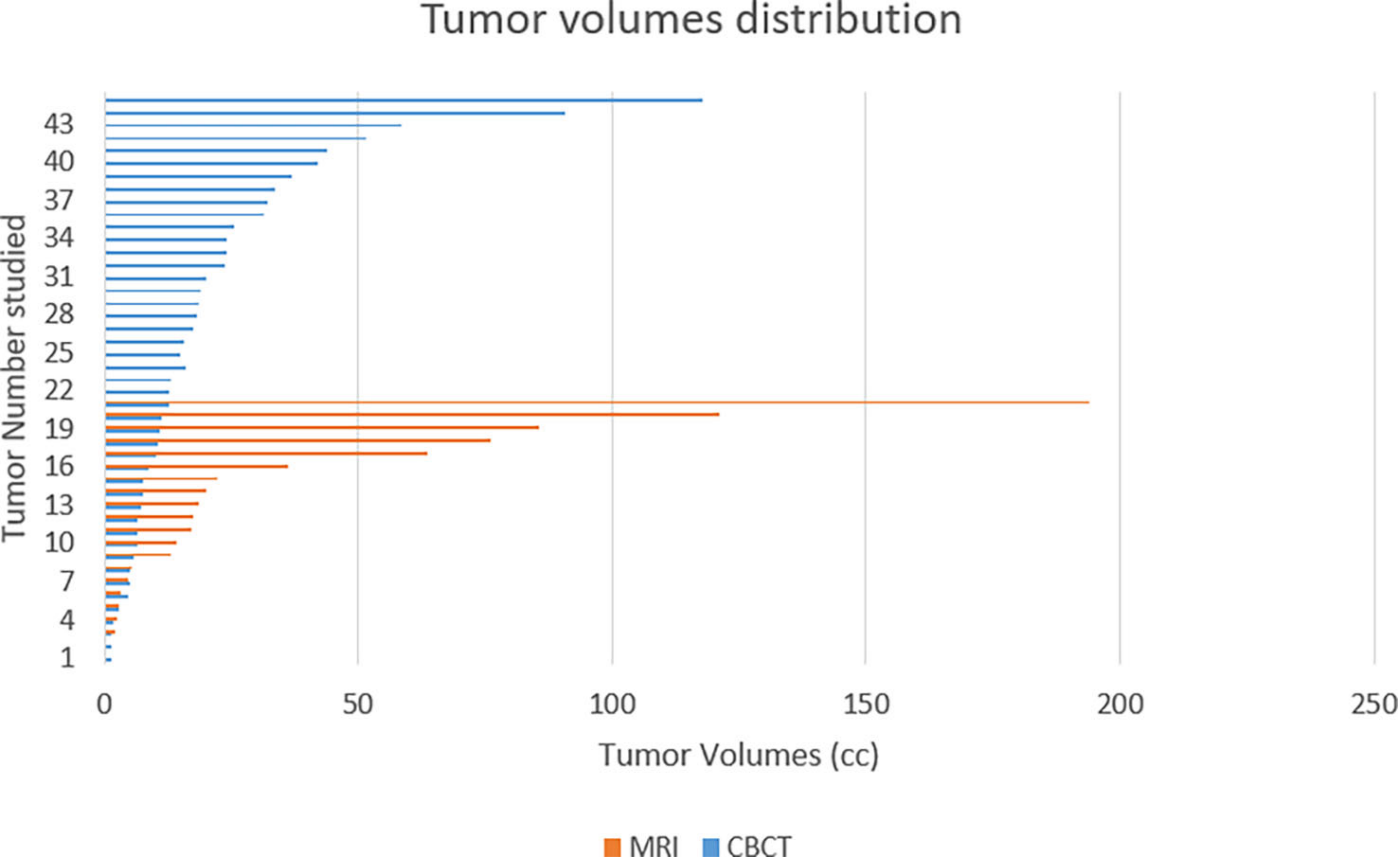
Patients treated using a conventional linac (CBCT) had non-significantly smaller tumor volumes than those treated on the MRIdian system (MRI).

The median and mean tumor volumes were 15.35cc and 25.92cc, respectively, ranging from 1.18 to 193.95cc. Patients in the CBCT group and the MRI group had a median tumor size of 13.03cc and 17.7cc, respectively (*p=0.467*) (**Figure 3**). The median PTV volume was 57.84cc and 44.25cc in the CBCT group and the MRI group, respectively. The median increase in target volume (by adding tumor margins as described above) was 74% and 60% in the CBCT group and the MRI group, respectively.

### Dosimetry parameters

**Table 3** details the comparative dosimetry metrics for both techniques. Despite a higher GTV in the MRI group, the mean healthy liver volume percentage receiving 15 Gy (V15Gy), and the healthy liver-mean dose, were 22.8% vs. 16.3% and 8.8 Gy vs. 6.3 Gy in the CBCT vs. the MRI group, respectively. Other metrics suggest similar PTV coverage with both techniques but a one third-reduction in healthy liver parenchyma irradiation by using MRgRT. Larger PTVs in the CBCT group undoubtedly accounted for these differences.

**Table 3:** Comparative dosimetry metrics for Linac- and MRIdian-based treatments.

Dosimetric parameter	VERSA-HD (45 pts)	MRIdian (19 pts)	p-value*
Healthy liver (Liver – PTV) dose			
Dmean (Gy), Mean (SD) Median	9.16 (3.57) 8.84	7.16 (3.64) 6.33	*0.034*
V15 (% healthy liver), Mean (SD) Median	22.76 (12.18) 18.73	16.27 (12.22) 12.14	*0.029*
Isodoses			
V40 (cc), Mean (SD) Median	96.71 (73.23)78.77	91.34 (104.82) 49.2	*0.151*
V30(cc), Mean (SD) Median	180.70 (126.05)133.02	152.68 (165.01) 92.89	*0.076*
V20(cc), Mean (SD) Median	396.59 (278.40) 294.22	294.61 (288.23) 190.7	*0.061*
V5(cc), Mean (SD) Median	2487.27 (1155.98)2240.33	2064.43 (1442.94) 1573.59	*0.079*
PTV coverage			
Dmean (Gy), Mean (SD) Median	46.36 (5.97) 45.31	44.95 (2.37) 44.44	*0.440*
Dmin (Gy), Mean (SD) Median	39.55 (7.01) 39.48	40.27 (2.04) 40.07	*0.121*
Dmax (Gy), Mean (SD) Median	49.88 (5.98) 48.38	48.36 (2.61) 48.4	*0.665*

*Wilcoxon test statistic

To directly compare treatment-planning system’s as well as machines’ performances, we recalculated theoretic dosimetry with the RayStation TPS for the VERSA-HD machine characteristics, using the same patient data (particularly the same PTV) as used in the MRIdian calculation, and compared the dosimetry metrics of the two treatment plans (see **Table 4**). The dosimetry metrics provided by the RayStation TPS and a VMAT technique appeared statistically significantly superior to those from the MRIdian system with its step-and-shoot IMRT method, as outlined by the conformation number (CN, median CN 0.90 vs. 0.83, *p=0.0017*), although other metrics were comparable.

**Table 4:** Comparative dosimetry metrics in patients treated on the MRIdian system, secondarily calculated for a fictive treatment on the VERSA-HD.

Test	Classes	Statistics	All (n=64)	VERSA (n=18)	MRI (n=18)	p-valeur (wilcoxon ou khi²)
Isodoses, V20		n	36	18	18	0.2417
		Mean (SD)	257.04 (268.35)	225.32 (243.20)	288.77 (294.92)	
		Median [Min-Max]	145.04 [22.10-1039.07]	134.17 [22.10-803.56]	177.70 [41.63-1039.07]	
Isodoses, V30		n	36	18	18	0.3113
		Mean (SD)	137.73 (154.36)	124.49 (142.64)	150.96 (168.33)	
		Median [Min-Max]	73.57 [10.78-553.25]	68.64 [10.78-464.36]	82.20 [17.96-553.25]	
Isodoses, V40		n	36	18	18	0.5478
		Mean (SD)	84.75 (101.57)	81.32 (98.92)	88.19 (106.91)	
		Median [Min-Max]	44.60 [5.63-356.43]	42.54 [5.63-330.88]	47.07 [6.52-356.43]	
Isodoses, V5		n	36	18	18	0.3930
		Mean (SD)	1786.38 (1422.04)	1642.59 (1373.06)	1930.16 (1494.75)	
		Median [Min-Max]	1377.45 [275.45-6362.34]	1377.45 [275.45-5927.95]	1411.91 [548.27-6362.34]	
Liver – PTV dose, Dmean		n	36	18	18	0.2681
		Mean (SD)	6.45 (3.56)	5.88 (3.43)	7.03 (3.70)	
		Median [Min-Max]	5.77 [1.44-16.02]	5.12 [1.44-13.81]	6.15 [1.82-16.02]	
Liver – PTV dose, V15		n	36	18	18	0.1737
		Mean (SD)	163.93 (128.37)	138.88 (112.57)	188.98 (141.15)	
		Median [Min-Max]	120.48 [31.01-597.71]	105.57 [31.01-443.70]	155.97 [38.41-597.71]	
Median CN		n	36	18	18	0.0017
		Mean (SD)	0.85 (0.08)	0.88 (0.06)	0.81 (0.08)	
		Median [Min-Max]	0.87 [0.68-0.95]	0.90 [0.70-0.95]	0.83 [0.68-0.93]	
PTV coverage, Dmax		n	36	18	18	<.0001
		Mean (SD)	49.49 (2.28)	50.98 (1.13)	48.00 (2.17)	
		Median [Min-Max]	50.27 [44.50-52.35]	51.22 [48.28-52.35]	48.38 [44.50-50.78]	
PTV coverage, Dmean		n	36	18	18	0.1210
		Mean (SD)	44.92 (1.55)	45.31 (1.50)	44.53 (1.53)	
		Median [Min-Max]	44.89 [42.06-47.69]	45.05 [43.02-47.69]	44.28 [42.06-47.09]	
PTV coverage, Dmin		n	36	18	18	0.0001
		Mean (SD)	39.60 (0.66)	39.37 (0.12)	39.84 (0.88)	
		Median [Min-Max]	39.45 [36.76-40.70]	39.38 [39.09-39.65]	40.07 [36.76-40.70]	

Hence, despite the superiority of the VMAT (using non-coplanar arcs) over the step-and-shoot method, both the tumor margins reduction and the gating function lead to similar target coverage with higher healthy tissue preservation.

### Local control and patients’ outcome

The median follow-up and imaging follow-up for all patients was 16.7 months [1.18-73.02] and 8.2 months [0.3-73.02], respectively. At last follow-up, 28 patients (47%) had died, 8 (14%) were lost to follow-up (and deemed deceased), whereas 23 (39%) were still alive. The local control rate was 76.6% (75.6% and 78.9% in the CBCT and MRI groups, respectively, *p=0.96*), meaning that 23.4% of patients experienced progression or relapse within the radiation field.

### SABR toxicity

The all-grade toxicity rate within the whole study population was 31.25% (33.3% and 26.3% in the CBCT and the MRI group, respectively). Grade ≥ 3 toxicity rate was 4.7% (4.4% and 5% in the CBCT and the MRI group, respectively). We observed one case of grade 5 radio-induced liver disease (in the CBCT group) in a hepatocellular carcinoma patient with a Child-score A6, who experienced decompensated cirrhosis with a Child-score C11. The only unexpected grade 3 toxicity observed in the MRI group was portal hypertension, occurring in a patient whose portal trunk received a total dose of 30 Gy in five fractions, in 7% of the volume (far below the dose constraint), and who had a thermos-ablation procedure in the segment I, potentially responsible for this adverse event. Noteworthy, ulcerous complications were less common in the MRI group (0 vs. 4.4% in the CBCT group; gastric hemorrhage, duodenal ulcer).

## Discussion

Over five years, 59 patients with liver tumors underwent SABR treatments in our department. This population represents a small sample size, which is explained by the routine use of competing treatments such as liver surgery, chemoembolization, and radiofrequency. Patients referred for liver SABR were therefore heavily pretreated, and more than half the patients previously received liver tumor-directed therapy, regardless of the group considered (**Table 2**).

We compared two radiation therapy techniques, using either a conventional Linac with a CBCT as the IGRT tool or an MR-Linac. The issues encountered with the use of CBCT as the IGRT tool are the need for fiducial markers implantation, the inability to differentiate the tumor from the healthy liver parenchyma, leading to increased tumor margin widths, which may limit further stereotactic treatments at the onset of new liver lesions. By directly visualizing and gating the tumor throughout the treatment session on the MRIdian system, we were able to both reduce the tumor margins and avoiding the invasive step of fiducial markers. The addition of security margins to the tumor led to a 74% and 60% increase of the target volume in the CBCT and the MRI group, respectively. Nonetheless, larger PTVs in the CBCT group come from both the non-visibility of the tumor and the non-use of the gating method, the latter likely participating the most in the volume expansion. However, the liver is subject to huge deformations, not only in the three main axes but also to some contractions and expansions. Hence, referring to the position of fiducial markers is a second-best and does not guarantee the exact location of the tumor, although some authors reported the successfully-use of a 5mm-margin added to the GTV, using the Exactrac Adaptive Gating Technique (5). This latter technique not only requires an invasive step but also assumes that the fiducial markers are strictly inserted within or close to the edge of the tumor and do not migrate to accurately reflecting tumor motion during the fraction. Hernando-Requejo et al. reported a 2-year local control rate of 87.3% by delivering a variable total radiation dose according to the tumor type (the median tumor size was not mentioned) (5). Thus, considering all the available radiation therapy techniques, the added value of the MRIdian system is to cumulate both the direct and clear visualization and gating of the tumor itself throughout the treatment delivery, as well as accurate tumor delineation at the time of treatment planning, without the need for image registration in the majority of cases. The precise tumor identification in three-fourth of the cases even allowed the reduction of isotropic tumor margins to 3 mm. Further reduction of tumor margins is not feasible because of MRI geometric distortion leading to a millimetric tumor-position uncertainty. Improvement of tumor visualization on MRI might occur from the use of a gadolinium-based contrast agent administered a few times before the SABR session (6). Moreover, new generation Gadolinium-based nanoparticles proved to be theranostic in *in-vitro* and *in-vivo* experimental studies (7, 8) and are currently examined in combination with whole-brain irradiation in patients with multiple brain metastases in a phase I study (9). It would be interesting to investigate its value and, above all, safety in MR-guided liver SABR treatments.

The MRIdian system is limited to step-and-shoot IMRT, using only coplanar beams, which might be sub-optimal, compared to VMAT treatments, in target coverage and organs at risk avoidance. Dosimetry metrics comparison showed similar target coverage but improved healthy tissue sparing when using MRgRT (**Table 3**). Hence, margins shrinking and direct tumor gating overcome the superiority of VMAT treatments (**Table 4**). On the other hand, treatment time was significantly longer in the MRI group, not impairing the value of ART, as investigated by Henke et al. (10).

MRgRT is new in the radiation therapy landscape and raised some questions about its safety as well as accuracy due to several new parameters to consider, such as the electron return effect (negligible in the liver), MRI to radiation therapy isocenter exact match, uncertainties with voxel size and tracking, MRI geometric distortion, and increased treatment time. Although positively addressed by the manufacturer, these uncertainties worth control of both unexpected toxicities and geographic missing and, therefore, lower local control rates. Grade 3 toxicity was uncommon, with only one patient affected in each group. However, portal hypertension experienced by the MRI-group patient is assumed to be likely thermo-ablation-induced. Gastritis and ulcerous disease were encountered only in patients treated on the VERSA machine; it likely constitutes a crucial strength of the MRIdian system attributable to tumor direct visualization and gating throughout the treatment session. In patients with a liver tumor close to radiosensitive organs, we were able to daily reshape the dose distribution, skirting around organs at risk. These results are in line with those observed by Henke et al. (11), who demonstrated in a phase I study that Stereotactic MR-guided online Adaptive Radiation Therapy (SMART) was able to break free from the 69% rate of dose constraints violation that occurred without plan adaptation, and allowed for no grade 3+ toxicity. Similarly, Rosenberg et al. (12) reported minimal grade 3 gastrointestinal toxicity (2 patients out of 26, 7%), slightly higher than what we found in our series, likely due to the lack of ART use. In our series, an adaptive plan was required in 20% of fractions in patients suitable for adaptive radiation therapy; however, the small sample size didn’t allow us to go deeper into this discussion.

The overall local control rate was 76.6% and was not worse by reducing tumor margins in the MRI group. It appeared in the range of local control rates published in the literature, closely linked to tumor size (13). Moreover, the local tumor control was not impaired by the higher median tumor size observed in the MRI group.

The weaknesses of this study mainly come from its retrospective nature, as well as its small sample size. Therefore, some significant biases may have influenced these results (14). However, patient characteristics appeared similar in the two groups, except for median tumor size (higher in the MRI group). Hence, patients outcomes have to be considered with caution, and the objective of reporting local control and toxicity was mainly to ensure the absence of both unexpected toxicity and worse tumor control. However, dosimetry metrics were prospectively collected and showed better healthy tissue preservation. These results, combined with better physicians’ confidence due to direct tumor visualization, both led us to the adoption of the MRIdian system for liver SABR.

## Conclusion

The use of MRI as IGRT allows for the reduction of the amount of healthy liver parenchyma irradiated without any decrease of the tumor control rate, which would be helpful for dose escalation (SMART method) or subsequent liver tumor irradiation if needed. Tumor margin reduction and gating also appear to reduce gastrointestinal toxicity.

## Data availability statement

The raw data supporting the conclusions of this article will be made available by the authors, without undue reservation.

## Ethics statement

The studies involving human participants were reviewed and approved by Clinic’s project selection group. The patients/participants provided their written informed consent to participate in this study.

## Author contributions

AT and JD have written the manuscript texts and performed the data collection. J-MB has prepared all figures and tables and has performed statistical analyses. All co-authors have reviewed and critiqued the manuscript. All authors contributed to the article and approved the submitted version.
